# Postulated Adjuvant Therapeutic Strategies for COVID-19

**DOI:** 10.3390/jpm10030080

**Published:** 2020-08-05

**Authors:** Anderson O. Ferreira, Hudson C. Polonini, Eli C. F. Dijkers

**Affiliations:** Fagron. Lichtenauerlaan 182, 3062 Rotterdam, The Netherlands; hudson.polonini@fagron.com (H.C.P.); eli.djikers@fagron.com (E.C.F.D.)

**Keywords:** COVID-19, SARS-CoV-2, coronavirus, dietary supplement, therapeutics, pharmacology, adjuvant, immunological, compounding, drug

## Abstract

The number of COVID-19 patients is still growing exponentially worldwide due to the high transmissibility of the SARS-CoV-2 virus. Therapeutic agents currently under investigation are antiviral drugs, vaccines, and other adjuvants that could relieve symptoms or improve the healing process. In this review, twelve therapeutic agents that could play a role in prophylaxis or improvement of the COVID-19-associated symptoms (as add-on substances) are discussed. Agents were identified based on their known pharmacologic mechanism of action in viral and/or nonviral fields and are postulated to interact with one or more of the seven known mechanisms associated with the SARS-CoV-2 virus: (i) regulation of the immune system; (ii) virus entrance in the cell; (iii) virus replication; (iv) hyperinflammation; (v) oxidative stress; (vi) thrombosis; and (vii) endotheliitis. Selected agents were immune transfer factor (oligo- and polypeptides from porcine spleen, ultrafiltered at <10 kDa; Imuno TF^®^), anti-inflammatory natural blend (*Uncaria tomentosa*, *Endopleura uchi* and *Haematoccocus pluvialis;* Miodesin^®^), zinc, selenium, ascorbic acid, cholecalciferol, ferulic acid, spirulina, N-acetylcysteine, glucosamine sulfate potassium hydrochloride, trans-resveratrol, and maltodextrin-stabilized orthosilicic acid (SiliciuMax^®^). This review gives the scientific background on the hypothesis that these therapeutic agents can act in synergy in the prevention and improvement of COVID-19-associated symptoms.

## 1. Introduction

The novel coronavirus syndrome (COVID-19), caused by the severe acute respiratory syndrome coronavirus 2 (SARS-CoV-2), was first reported in Wuhan (China) and has spread worldwide. The number of infected humans is growing exponentially due to the high transmissibility of the virus. COVID-19 has two major predecessors: Severe Acute Respiratory Syndrome (SARS) and Middle East Respiratory Syndrome (MERS), firstly described in 2002 and 2012, respectively [1].

The scientific community has been making significant efforts globally to elucidate the mechanisms of action of SARS-CoV-2, to understand the predisposition of severe cases, and to find possible treatment options. The focus for the research of therapeutic agents currently includes antiviral drugs, vaccines, and other adjuvants agents that could relieve symptoms or improve the healing process. For the latter, scientists have been focusing their interests on a long list of essential nutrients, herbal extracts, phytochemicals, and other nutraceuticals that might either help to prevent virus entrance in the cells or to possibly decrease its replication rate [2,3]. The use of nutraceuticals can also be taken into account within the context of prophylaxis, which is for special interest of high-risk or highly exposed population subgroups, such as the elderly and healthcare personnel, respectively.

Nutraceuticals for prophylaxis are often aimed at improving immune system function, which is important for infectious diseases in general and SARS-CoV-2 in particular. It is believed that decreased immunity is at least partially responsible for the observed increase in morbidity and mortality resulting from infectious agents, including SARS-CoV-2, particularly in the elderly [4]. Dietary supplementation with specific nutrients that can improve the immune defense has been gaining momentum in current days as it is well-established that the nutritional profile can influence the patient’s immunity [5].

Based on extensive study of the SARS-CoV-2 infection pathways and the parameters involved in the different stages of the disease, we here describe a set of 12 therapeutic options that could act synergistically and play a role in the prevention and/or treatment of the symptomatology associated with COVID-19, detailing their possible mechanisms of action.

## 2. SARS-CoV-2 and COVID-19

The coronaviruses (order *Nidovirales*, family *Coronaviridae*, and subfamily *Orthocoronavirinae*) are spherical (about 125 nm in diameter), nonsegmented, and enveloped viruses with club-shaped spikes on the surface giving the appearance of a crown, or “solar corona” [6]. They are single-stranded RNA viruses and generally circulate among animals. The betacoronavirus genus includes MERS-CoV (Middle East Respiratory Syndrome-virus), SARS-CoV (Severe Acute Respiratory Syndrome-virus), and SARS-CoV-2 (Severe Acute Respiratory Syndrome 2-virus). The SARS-CoV-2 is spherical (60–200 nm in diameter) and normally contains four proteins: spike glycoproteins S, E, M and N.

Human infection begins when the virus enters the host cell. To date, the best known cell membrane entry receptor for SARS-CoV-2 is the angiotensin-converting enzyme-2 (ACE-2), which uses the cellular transmembrane serine protease 2 (TMPRSS2) for S protein priming [7]. This in turn leads to the fusion of the viral and host cell membranes through the endosomal pathway and SARS-CoV-2 RNA is released into the host cell cytoplasm. Inside the human cell, replication is performed by RNA-dependent RNA polymerase (RdRP) and newly produced virions are released from the host cell by exocytosis, amplifying the process [7,8,9]. Respiratory epithelial cells, located in the respiratory tract (nasal cavity, larynx, bronchi, bronchioles, and alveoli) are the preferred cells for replication of the SARS-CoV-2 virus [10]. However, the ACE2 receptor is also found in different extrapulmonary tissues, including heart, kidney, and intestine, which could explain these organs being compromised in COVID-19 [11].

### 2.1. Clinical Features of the SARS-CoV-2 Infection

Human-to-human transmission of the SARS-CoV-2 infection can occur through the contact of nasal and oral mucosa with aerosol or droplet particles from symptomatic or asymptomatic patients or via direct inoculation of the respiratory epithelium through the hands [6,12]. The surface has been suggested as a site of infection, but there is not enough evidence to date whether this is prone to occur [13]. It has been shown that SARS-CoV-2 can remain viable in aerosols for up to 3 h; on surfaces, it was found to be more stable in plastic and stainless steel, with viable viruses detected for up to 72 h (mean half-life of 5.6 h on steel and 6.8 h on plastic)—while no viable viruses could be found after 4 h on copper and after 24 h on cardboard [14,15]. SARS-CoV-2 RNA can be found in nasopharyngeal swabs, feces, blood and (very rarely) in urine [16,17].

The average incubation period of SARS-CoV-2 is about 4–6 days, with about 95% of cases developing symptoms in the 14 days following infection [18,19], i.e., the patient can then be asymptomatic but still transmitting the virus [20,21,22,23]. Additionally, although the viral load drops after the first week, a prolonged increase can be observed up to 37 days [23,24]. It is therefore not exactly known when the patient stops shedding the virus and is no longer contagious.

The most common symptoms of COVID-19 are fever, dry cough, systemic fatigue; less common symptoms are runny nose, sore throat, sneezing and shortness of breath (around the seventh day after symptom onset). Other clinical manifestations (rare in most patients) may include: myalgia, gastrointestinal symptoms (diarrhea), impaired renal function, neurological symptoms (e.g., viral encephalitis), olfactory and gustatory dysfunctions (e.g., anosmia, dysgeusia), cardiologic conditions (e.g., myocarditis); and dermatologic findings (e.g., widespread urticaria, erythematous rash, and frostbite toes) [24,25].

Vulnerable groups for developing severe COVID-19 include the elderly, patients with co-morbidities (cardiovascular disease, hypertension, diabetes, chronic respiratory disease, chronic kidney disease undergoing dialysis, liver disease, cancer), obese, smokers and immunocompromised patients [26,27]. Children and younger adults usually present a more benign syndrome, possibly related to a lower expression of ACE2 receptor in the pediatric population [28]. Furthermore, gender may also affect ACE2 expression: ACE2 levels are higher in men than in women, which could partially account for the higher severity and mortality among men patients [28,29].

### 2.2. COVID-19 Stages

**Figure 1:**
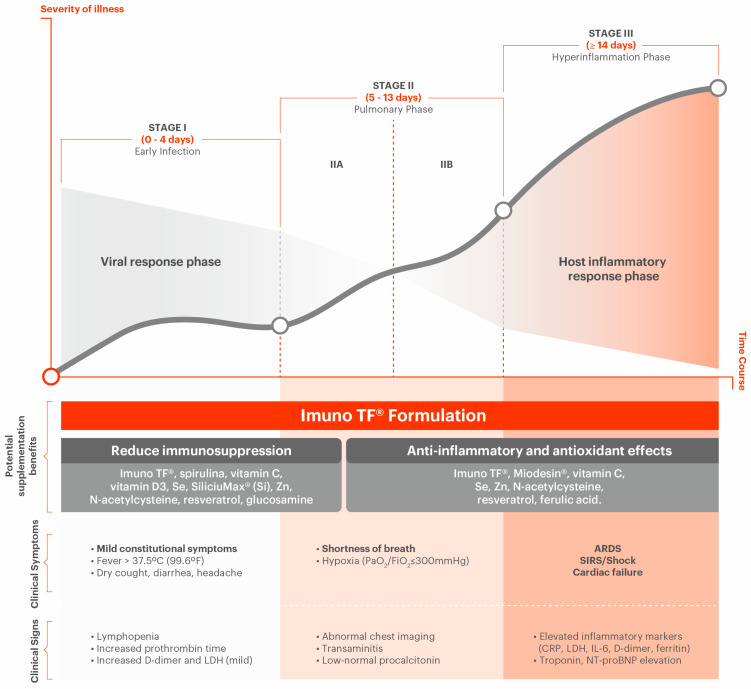
Time course of COVID-19 through the different stages of the infection, and the possible effect of the selected therapeutic agents in different stages of the syndrome. Adapted from Siddiqi [30]. ARDS: Acute respiratory distress syndrome. SIRS: Systemic inflammatory response syndrome. CRP: C-reactive protein. LDH: Lactate dehydrogenase. NT-proBNP: N-terminal-pro hormone BNP (brain natriuretic peptide).

One recent study has proposed the use of a three-stage classification system for COVID-19, taking in consideration the increasing severity of the syndrome and their distinct clinical findings and response to therapy [30]. A scheme of the three stages is shown in Figure 1.

*Stage I: Mild (early infection, viremia phase)*

The initial stage occurs at the time of viral innoculation and incubation (viral response phase). The symptoms are typically nonspecific (e.g., cough, fever, diarrhea). Diagnosis at this stage includes respiratory tract sampling (polymerase chain reaction, PCR), serum testing for SARS-CoV-2 IgG and IgM, chest imaging, complete blood count (lymphopenia and leukopenia), and liver function tests. Treatment at this stage should focus on symptomatic relief. If applied, antiviral therapy is aimed at reducing the duration of symptoms, minimize contagiousness, and prevent progression of disease [30].

*Stage II: Moderate (Pulmonary Involvement with and without Hypoxia; pneumonia phase)*

This stage is represented by the establishment of pulmonary disease (pulmonary phase), viral multiplication, and inflammation in the lung. Patients can develop viral pneumonia, with cough, and maybe hypoxia (PaO_2_/FiO_2_ < 300 mmHg). Imaging (Chest X-Ray and/or Computed Tomography) can reveal bilateral infiltrates or ground glass opacities. Blood tests can show increasing lymphopenia, along with transaminitis. Procalcitonin is typically low to normal, unless comorbid bacterial infection is present [31]. Systemic inflammatory markers may be elevated. The patient at this stage should be monitored intensively. Treatment is focused on symptomatic measures: in Stage IIa patients (without hypoxia), corticosteroids should be avoided; in Stage IIb (with hypoxia), anti-inflammatory and antiviral therapy, together with mechanical ventilation, can be considered as necessary [30].

*Stage III: Severe (Systemic Hyperinflammation, Severe or Recovery phase)*

This stage develops when extrapulmonary, systemic hyperinflammation occurs (e.g., a cytokine storm). In this stage, inflammatory biomarkers (e.g., IL-2, IL-6, IL-7, granulocyte-colony stimulating factor, macrophage inflammatory protein 1-a, TNF-a, CRP, ferritin, and D-dimer) are elevated. Troponin and N-terminal proB-type natriuretic peptide (pro BNP) can also be elevated. Helper, suppressor, regulatory, CD4^+^ and CD8^+^ T cells are decreased; in fact, reduced functional diversity of T cells in peripheral blood may predict a severe progression in COVID-19 patients [32,33]. It has been postulated that the “cytokine storm” is a result of the lungs being infected by SARS-CoV-2, a suppressed immune response, elevated inflammation, and excessive oxidation stress. A form similar to hemophagocytic lymphohistiocytosis (HLH) can occur in patients in this advanced stage of the disease due to excessive immune activation, thus leading to further tissue damage [32,34]. Shock, vasoplegia, respiratory failure and even cardiopulmonary failure are detectable at this stage. The involvement of systemic organs (e.g., myocarditis) can manifest during this stage. The therapy at this stage can include the use of immunoregulatory agents to reduce systemic inflammation before it results in multiorgan dysfunction [32].

An important feature of the COVID-19 syndrome is that acute lung injury (ALI)/acute respiratory distress syndrome (ARDS) may ensue, accompanied by a series of complications, the outcomes of which vary according to the severity of the disease [34,35,36,37]. ARDS characteristically is accompanied by a rapid onset fibrosis, which can be understood not only as a comorbidity, but also as one main player in the mortality of COVID-19, and the fact that IL-6 (the proinflammatory cytokine that is involved in connective tissue disorders, fibrosis included) is found increased in patients who died (and not in the ones that recovered from the syndrome) corroborates this theory [38]. The pulmonary fibrosis gains relevance because it is a finding that normally follows chronic inflammation (observed in COVID-19 severe cases) or is an idiopathic pulmonary fibrosis (IPF), whose risk factors are increasing age, male sex, and comorbidities such as hypertension and diabetes (also risk factors for severity of the COVID-19) [39,40]. The severe fibroproliferative lung disease has also been associated with a prolonged need for mechanical ventilation [41]. Additionally, similarly to what was observed with MERS, the fibroproliferative process could be observed in older patients even after their recovery—therefore impacting their long-term quality of life [39,41,42]. In this sense, therapies with antifibrotic effects can both help in preventing patients with IPF from developing Stage III symptoms and in preventing COVID-19 without IPF from developing fibrosis during infection or after the recovery [40].

### 2.3. Complications Associated with COVID-19

In high-risk groups, the immune response can be not sufficient to eliminate the SARS-CoV-2 and consequently cure the patient. Here we elaborate on some of the key points that can induce more severe stages of the syndrome.

#### 2.3.1. Hyperinflammation Due to Immune System Overresponse (Cytokine Storm)

The antiviral responses of the host’s innate and adaptive immune system, involving the activation of T cells (CD4^+^ and CD8^+^) and the production of several proinflammatory cytokines, are essential for controlling viral replication. However, in some patients the viral tissue injury induces exaggerated production of proinflammatory cytokines, and the exacerbated recruitment of proinflammatory macrophages and granulocytes. Therefore, inhibition of cytokines, and IL-6 specifically, may help prevent severe lung tissue damage caused by cytokine release in COVID-19 patients [43,44].

#### 2.3.2. Immune Dysregulation

Regulatory T cells are responsible for the maintenance of the immune homeostasis, suppressing the activation, proliferation, and proinflammatory function of most lymphocytes, including CD4^+^ and CD8^+^ T cells, NK cells, and B cells (the latter two are known to be reduced in COVID-19 patients) [34]. This is in line with what is seen in COVID-19 patients: a significant lymphopenia (representative of an impairment of immune system) develops in most COVID-19 patients, especially in those with more severe disease. This immune dysregulation can be triggered by the SARS-CoV-2 through its effects on the subsets of T cells. The level of helper, cytotoxic suppressor and regulatory T cells decreases, and is even more noticeable in severe cases [33].

#### 2.3.3. Antibody Profile

The immunoglobulins (antibodies) profile may be an early predictor of the severity and prognosis of COVID-19. In a study investigating the dynamics of IgA, IgG, and IgM antibody responses in mild and severe SARS-CoV-2 infections, the researchers have found out that IgA antibodies could work as a diagnostic marker, as they start to circulate early after the mild COVID-19-associated symptoms [45]. In addition, there is evidence for recruitment of immune cells populations (antibody-secreting cells, T helper cells and activated CD4^+^ and CD8^+^ T cells), with IgM and IgG SARS-CoV-2 binding antibodies in the patient’s blood before COVID-19 symptoms are resolved [37,46,47].

#### 2.3.4. Hematologic Consequences

Recent studies underline that severe COVID-19 can be complicated by intravascular coagulation, especially when combined with long-term bed rest, and that this thromboembolism in patients with severe COVID-19 contributes to a significant percentage of the deaths from the disease [48]. In one meta-analysis, the hemoglobin value was found to be significantly lower in COVID-19 patients with severe disease than in those with milder forms of the disease [49].

#### 2.3.5. Endotheliitis

Endothelial cells form the barrier between vessels and tissues. They are found exclusively in vascularized tissue, forming a single layer of cells that covers the interior surface of blood and lymphatic vessels, and express ACE2 receptors. A recent case report has shown the presence of SARS-CoV-2 viral elements within endothelial cells, with an accumulation of inflammatory cells and some evidence of endothelial death. These findings have suggested that SARS-CoV-2 infection facilitates the induction of endotheliitis in multiple organs, as a consequence of viral involvement and of the host inflammatory response. Endotheliitis could explain the systemic impairment of the microcirculatory function in different vascular beds and their clinical sequelae in patients with COVID-19. The increase in vascular permeability can lead to infiltration of monocytes, macrophages and T cells, as well as the systemic cytokine storm and the pulmonary edema and pneumonia [50]. The endotheliitis hypothesis provides a rationale for therapies to stabilize the endothelium while tackling viral replication, particularly with anti-inflammatory and anti-cytokine drugs, and also ACE2 inhibitors and others endothelial barrier protectors [51,52].

Additionally, some neuropathological analysis from COVID-19 patients revealed that the SARS-CoV-2 infected Neuropilin 1 (NRP-1) positive cells in olfactory epithelium and bulb, as spike proteins were detected in those cells. This finding suggests the existence of an NRP-dependent intranasal brain entry pathway and, indeed, NPR1 is highly expressed in endothelial cells and in the epithelial cells facing the nasal cavity. In this case, NPR1 would also serve as an entry factor, not only ACE2, which could explain the enhanced tropism of SARS-CoV-2. In this light, vascular endotheliitis, thrombosis, and angiogenesis, together with the upregulation of NRPs in SARS-CoV-2 infected blood vessels, should be taken in consideration for the determination of prognostic and treatments of the COVID-19 syndrome [53,54].

## 3. Therapies for Potential Prevention and Improvement of the Associated Symptoms

Based on extensive study of the SARS-CoV-2 infection pathways and the parameters involved in the different stages of the disease, we have identified 12 agents that could play a role in the prevention and/or support treatment of the symptomatology associated with COVID-19 (Table 1). The therapy can be tailored to the patient’s needs or given all together as a single formulation.

### 3.1. Transfer Factors

Transfer factors (TF) are immune structures composed of oligo- and polypeptides fractions, obtained from various sources. Commercially available traditional TF extracts are generally obtained from colostrum (the first milk produced by mammals), bird’s egg yolks, or porcine spleen [55,88]. The origin of the TF is essential for the final quality and safety of the product. As the role of colostrum is to provide to the newborn’s immune system initial protection [89], it contains immunoglobulins, that are known to be able to cause allergic reactions [90]. Purified spleen extracts, on the contrary, are generally purified to avoid elicit an immune response [91].

TF were first described in 1955 by Henry Sherwood Lawrence and were later characterized at the molecular level by Kirkpatrick [92,93,94]. In his study, Lawrence demonstrated that a dialysis of leukocyte extract from a healthy donor, presenting a positive response to the percutaneous tuberculin test, was able to transfer to a healthy recipient the ability to also respond positively to this test [92]. In 1983, Lawrence and Borkowsky modified the original TF purification protocol using a dialysis membrane and a second molecular exclusion membrane to obtain molecules with three different sizes: <3.5 kDa (which would contain molecules such as serotonin, histamine, bradykinin, ascorbate, nicotinamide etc.), >3.5 kDa, and <12 kDa. The fractions between 3.5 kDa and 12 kDa had the ability to bind to antigens [95].

The TF could then be understood as oligoribonucleotides attached to a peptide molecule inherent in all animal organisms [96,97]. These molecules appear to be produced by T helper cells and formed by short chains of amino acids with small pieces of ribonucleic acid (RNA) [89,98]. The attached portion of RNA is probably related to a cytophilic property and the specificity of the TF [55]. In fact, biochemical analyses suggested that the biological activity of TF was the result of the oligoribonucleotide linked to the amino termination of peptide, as its absence resulted in no activity [99]. Kirkpatrick identified a highly conserved region of amino acids in the TF with the ability to bind to target cells with high affinity [58]. There are reports of around 18 amino acids present in TF (the reason why they are considered oligo- or polypeptides) [100,101], and high tyrosine and glycine contents are present in some variants [55]. In fact, TF as a product is a complex extract, containing a high number of different TF molecules, and not just a single chemical entity—and each of these molecules has its own specific function and purpose in a regulated immune system [88]. In addition, they are apparently not species-specific, i.e., TF produced in one species is effective in another animal species [89,102].

**Figure 2:**
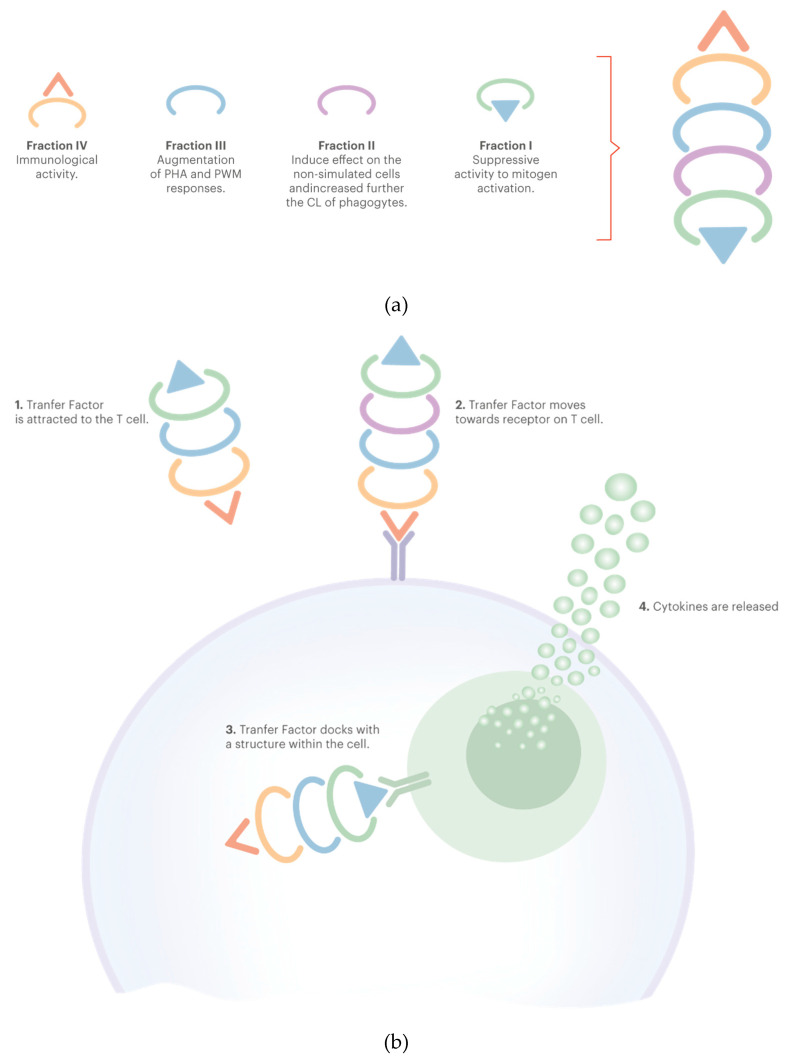
(**a**) Structure of transfer factor fractions. (**b**) Schematic representation of the induction of immune response by transfer factor. Adapted from Salazar-Ramiro et al. [57].

TF contains two subunits within its molecule: tuftsin (peptide fraction) and splentopentin, providing immune system-enhancing activity. Tuftsin is believed to be the subunit that stimulates the production of macrophages [103]. The biologically active fraction of human TF has been separated by exclusion chromatography in some subcomponents (this has been summarized in Figure 2). The suppressor activity is contained in Fraction I. Fraction II has a direct chemiluminescence (CL) inductive effect on nonstimulated cells and increases CL of phagocytes. Fraction III contains components responsible for increase in the phytohemagglutinin (PHA) and pokeweed mitogen (PWM) responses. Fraction IV would be responsible for the immunological activity itself [57].

The natural immune response is the causal factor for the in vivo production of TF, as they are produced in T helper cells. After the TF are released, the activity of the immune system is influenced in several pathways. The presence of TF is understood by the other cells involved in the immune system as an indication that T helper cells (Th1 immunity) are active in the mechanistic elimination of the pathogen, thus stimulating the production of new T helper cells, natural killer (NK) cells, macrophages, and cytotoxic T cells, and also the conversion of young lymphocytes into Th1 immune cells. The increase of Th1 cells, for its turn, suppresses the production of Th2 cells and their related cytokines, such as IL-4, IL-5, IL-6 and IL-13, while there is an increase in Th1-related cytokine levels (essentially IFN-γ) and a general strengthening of the Th1 response [55,91]. It is possible that TF influence key aspects of immune function by stimulating the related Th1 immune cells to release cytokines, thus influencing subsequent immune activity. Kirkpatrick (1989) measured levels of various cytokines after oral administration of TF to human subjects. Among the cytokines, the author found increased levels of IFN-γ, which is relevant since it is produced only by Th1 cells, cytotoxic T cells and NK cells. Thus, it indicates specificity of TF for activation of the Th1 response mechanism [104]. INF-γ can inactivate viruses and promote differentiation of young WBC into Th1 cells, which partially explains how TF leads to the recruitment of new cells for Th1 immune responses [91]. A possible inhibition of the production of TNF-α by monocytes and of the NF-kB activity in human T cells was also reported [57].

A peculiar feature of TF is the elicitation of multiple opposite functions: specific antigen fractions help the recognition of pathogenic micro-organisms, which increases the antigenic stimulus. Oppositely, TF also suppresses Th2 cells through the release of IL-10, thus playing a role in controlling excessive immune reactions [55]. In this sense, TF can possibly regulate the immune system by stimulating it against threats (micro-organisms or tumor cells), while avoiding immune hyperresponsiveness and autoimmune reaction.

The most striking example of TF application has been shown as early as 1980. Sixty-one patients with leukemia and no immunity to chickenpox were given TF or placebo and followed for 12 to 30 months in a double-blind trial designed to examine the clinical efficacy of TF. Sixteen patients in the transfer-factor group and 15 in the placebo group were exposed to varicella zoster, and most of them had a rise in antibody titer. Chickenpox developed in 13 of 15 exposed patients in the placebo group but in only one of 16 in the TF group (P = 1.3 × 10^−5^). In the patients treated with TF and exposed to varicella without acquiring chickenpox the titer of antibody to varicella zoster was equal to that in the patients given placebo who became infected with chickenpox [105].

To the best of the authors’ knowledge, there is no randomized clinical trial evaluating the role of transfer factors standalone for COVID-19.

### 3.2. Anti-Inflammatory Natural Blend

Miodesin^®^ is a co-processed, anti-inflammatory blend of natural origin composed of registered proportions of *Uncaria tomentosa*, *Endopleura uchi* and *Haematococcus pluvialis*. It has been developed to act in clinical conditions in which the inflammatory process plays a major role (for instance, infectious diseases with important inflammation findings, osteoarthritis and joint health, endometriosis, uterine leiomyomas, adenomyosis, and fibromyalgia), whether for oral or intravaginal use [106,107].

*Uncaria tomentosa* (Willd.) DC. (Rubiaceae) (popular name: cat’s claw) possesses indole and oxindole alkaloids, glucosinolates, flavonoids, sterols, carbolines and polyunsaturated fatty acids, which confer immunostimulant, antioxidant and anti-inflammatory activities [108]. The anti-inflammatory properties seem to be mediated by mitraphylline, via inhibition of IL-1α, 1β, 17, and TNF-α [109]. Moreover, *U. tomentosa* can decrease lipid peroxidation and reduce the level of reactive oxygen species (ROS) [110]. The plant extracts also showed moderate activity against bacterial (*Bacillus cereus*, *B. subtilis*, *Enterococcus faecalis*, *Staphylococcus aureus*, *S. epidermidis*, *Escherichia coli*, *Mariniluteicoccus flavus*, *Streptococcus mutans* and *Klebsiella pneumoniae*) and fungal strains (*Candida albicans*) [111]. There’s evidence that *U. tomentosa* can decrease inflammation and regulate immune system through the extension of lymphocyte survival via an anti-apoptotic mechanism [112] and the inhibition of the production of TNF-α, a critical cytokine in chronic inflammation conditions, due to its regulation role on the activation of activator protein 1 (AP-1) and release of NF-κB [113,114].

*Endopleura uchi* (Huber) Cuatrec. (Humiriaceae) is a tree found throughout the entire Brazilian part of the Amazon basin. Traditional medicinal applications of the stem bark of *E. uchi* are diverse and include anti-inflammatory and antibacterial activities. Antifungal activities against *C. albicans*, *C. tropicalis* and *C. guilliermondii* [115] were also reported. *E. uchi*, presents anti-inflammatory activity through the selective inhibition of cyclooxygenase-2 (COX-2) and phospholipase A2 (PLA2) provided by its major compound, bergenin [116]. Bergenin also downregulates the phosphorylation of NF-κB and mitogen-actived protein kinases (MAPK), resulting in decreased infiltration of inflammatory cells, and decreased levels of nitric oxide (NO), TNF-α and IL-6 [117].

Finally, *Haematococcus pluvialis* (Chlorophyceae, Volvocales) is an unicellular fresh water microalga, considered to be the main source of astaxhanthin [118]. Astaxanthin (3,3′-dihydroxy-ß-carotene-4,4′-dione) is often referred as a “super-antioxidant” molecule, because of its capacity to reduce free radicals and oxidative stress: 65 times greater than vitamin C, 54 times more than β-carotene, and 100 times more α-tocopherol—and the synthetic molecule has 20 times lower antioxidant capacity than its natural counterpart [119]. Additionally, the molecule also presents immunomodulating activity, demonstrated both in animal and human studies [120]. Studies have shown that it can reduce lipid peroxidation and protect LDL from oxidative inducers [121,122]. The antioxidant activity is proposed to occur due to an equilibrium with the enol form of the ketone, when the molecule is in solution. This would result in a dihydroxy conjugated polyene system, which presents a hydrogen atom that could break the free radical reaction [123,124]. It can also be considered an endothelial protector due these antioxidant properties: it has been demonstrated to inhibit intracellular induced stress in human endothelial cells without any cytotoxicity and modification of the cell morphology [125].

Additionally, a recent study has shown that Miodesin^®^ has its potent anti-inflammatory activity due to the fact that its components can act synergistically inhibiting hyperactivation of chondrocytes, keratinocytes, and macrophages, through the decrease in the release of cytokines (IL-1β, IL-6, IL-8, and TNF-α) and chemokines (CCL2, CCL3, and CCL5) and the expression of NF-κB, inflammatory enzymes (COX-1, COX-2, PLA2, iNOS), and chemokines (CCL2, CCL3, and CCL5) [61].

To the best of the authors’ knowledge, there is no randomized clinical trial evaluating the role of Miodesin^®^ standalone for COVID-19.

### 3.3. Potential Activities of the Ingredients in COVID-19 Pathophysiology

**Figure 3:**
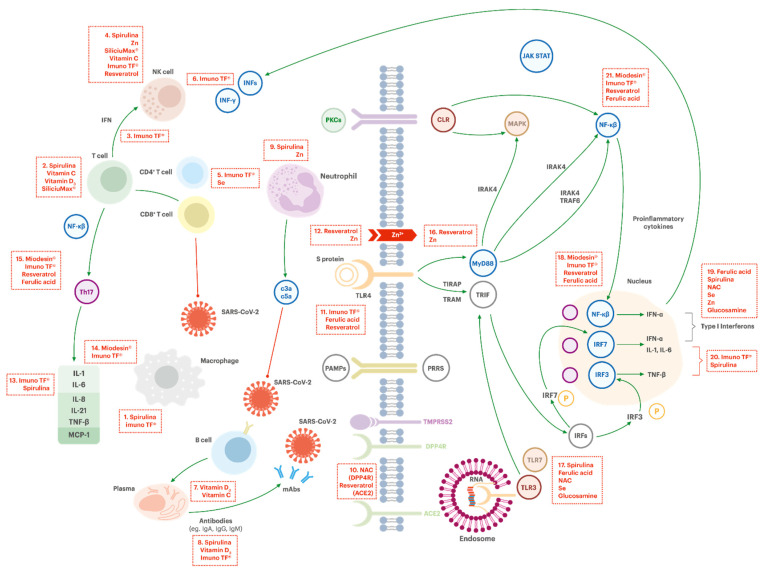
Innate immune response and adaptative response of coronaviruses infections and potential targets for immunoregulation by selected therapeutic agents. Adapted from Dosch et al. [126].

**Figure 4:**
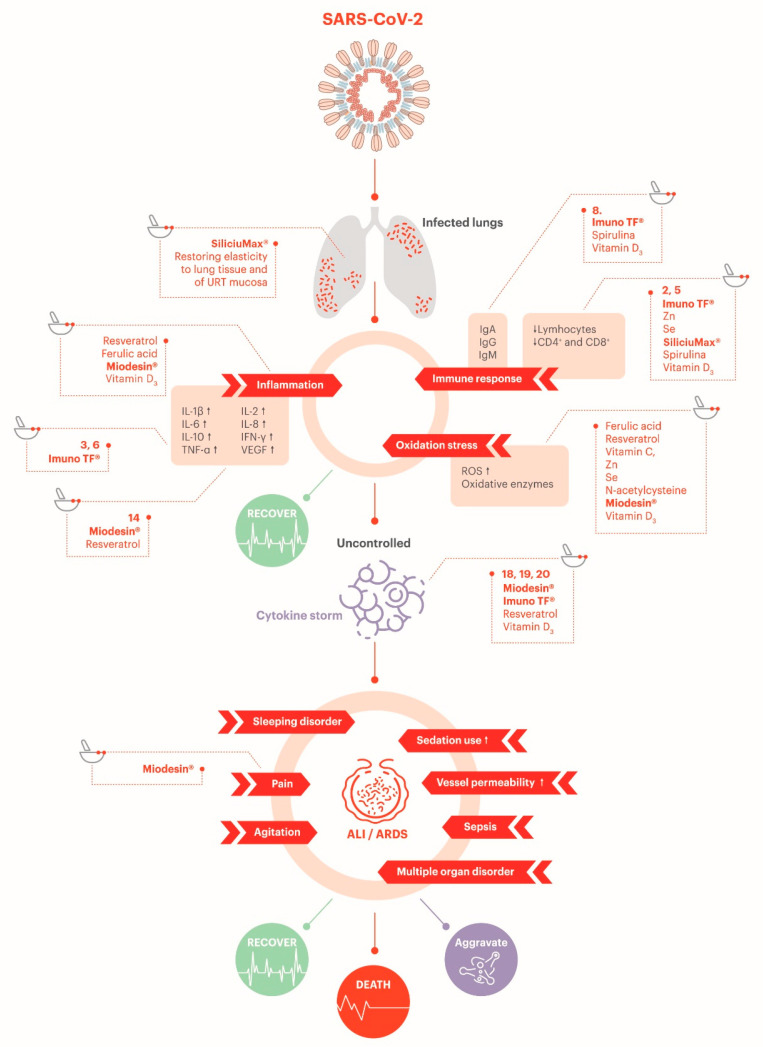
Main effects known to date of SARS-CoV-2 on human organism and sites of action. URT: Upper respiratory tract. ALI: Acute lung injury. ARDS: Acute respiratory distress syndrome. Adapted from Zhang et al. [36].

The mechanism of action of each component is illustrated in Figure 3 and Figure 4, which depicts the general mechanism by which the coronaviruses can infect a human cell and highlights the potential targets.

#### 3.3.1. Regulation of the Immune System

##### Macrophage Activation (Box #1)

Macrophages are present in all tissues. They possess several functions in immune homeostasis, such as host protection, tissue repair, phagocytosis, and secretion of diverse factors for immune communication, which contribute to innate and adaptive defenses against infections and to counteract inflammatory processes [127]. The activation of macrophages involves the synergistic action of cytokines, chemokines, and pathogens-associated molecular patterns (PAMPs). TF regulate the antigenic stimulus, which causes the production of IFN-γ, the most potent macrophage-activating factor [55,104]. Spirulina supplementation can also play a role in macrophage activation. Studies show that spirulina stimulates the immune system by increasing phagocytic activity of macrophages—and this can lead to increased amounts of NK cells in tissues through the production of IL-12 and IL-15 by them [85,86].

##### Activation of NK Cells (Box #4)

NK cells are part of the first line of defense against infected cells (e.g., viruses and bacteria). Splentopentin from the TF and spirulina, zinc, orthosilicic acid, ascorbic acid, and trans-resveratrol can potentially increase the activation of NK-cells, improving immune response against foreign invaders [65,66,73,80,86,103,128].

##### Increasing T Cells Functions (Box #2)

In COVID-19, T cell counting and functionality are reduced [33]. T cells are central regulators of the immune response and exert their actions by modulating the function of other immune cells. Spirulina, ascorbic acid, cholecalciferol, and orthosilicic acid might potentially enhance T cells functions, promoting their activation and proliferation [68,71,87,129].

##### CD4^+^ Cells Activation (Box #5)

The activation and differentiation of CD4^+^ T cells play a critical role in establishing and subsequently controlling protective adaptive immune responses, such as activation of B cells, which produce antibodies, and CD8^+^ T cells, which are able to eliminate infected cells. TF improve the recognition of antigens that have entered the body and the formation of immune memory. TF and selenium can regulate the antigenic stimulus triggering CD4^+^ Th1 cells (T helper type 1 cells: CD4^+^ effector T cell that secretes IFN-γ, IL-2, IL-10, and TNF-α/β and promotes cell-mediated immune responses and is required for host defense against intracellular viral and bacterial pathogens) to produce IFN-γ, IL-1 and TNF-α [55,68,69,91,104]. Selenium can stimulate CD8^+^ lymphocytes, NK cells, and macrophage phagocytosis [69].

##### IFN-γ Production (Box #3 and #6)

Interferons (IFNs) are a group of proteins usually produced in response to viral infections, and it can recruit Th1 cells to the inflammatory site and downregulate the activity of Th2 cells (T helper type 2 cells: CD4^+^ effector T cell that secretes IL-4, IL-5, IL-9, IL-13, and IL-17E/IL-25 and are required for humoral immunity) [130]. The production and release of IFN-γ then acts on the neutrophils recruitment, which helps to control the infection and the consequent inflammation observed, and also in the development of the acquired immunity. It is also observed that severe COVID-19 patients present a lower expression of IFN-γ related to the decrease and impaired CD4^+^, CD8^+^, and NK lymphocytes [131]. In fact, a high IL-6/IFN-γ ratio seems to predict severe COVID-19 disease and lung damage due to the cytokine storm [132]. TF have the potential to regulate the antigenic stimulus, which can cause the production of IFN-γ by NK cells [55,104].

##### Antibodies Production (Box #8)

Spirulina, cholecalciferol and ascorbic acid potentially modulate circulating lymphocytes, and influence the antibodies levels [68,85].

##### Development and Function of Neutrophils (Box #9)

Spirulina and zinc may increase the rate of production and development, as well as enhancing the function of cells mediating nonspecific immunity, such as neutrophils [68].

##### Improving Tissue Barrier Function on Innate Immunity

It is suggested that orthosilicic acid and ascorbic acid are important for activating the hydroxylation enzymes, which are important to the formation of the collagen network, improving skin strength and elasticity. They are also cytoprotective agents and relevant nutrients for extracellular matrix of the skin and mucosa, optimizing their roles on innate immunity against infections—i.e., it could potentially strengthen the mucosal barrier to prevent SARS-CoV-2 penetration [133,134,135].

#### 3.3.2. Support on Avoidance of Virus Entrance in the Cell

##### Reducing the Virus Entrance in the Cell (DPP4R Inhibitory Effect, ACE2 Blocker Effect) (Box #10)

There are currently three exopeptidase receptors known for coronaviruses in human cells: ACE2 (angiotensin conversing enzyme 2), APN (aminopeptidase N) and DPP4 (dipeptidyl peptidase-4, also known as adenosine deaminase complexing protein 2). DPP4 mRNA and protein expression are inversely correlated with lung function and diffusing capacity parameters, which can partially explain the fact that smokers and chronic obstructive pulmonary disease (COPD) patients seem to be more susceptible to coronaviruses. N-acetylcysteine and natural polyphenolic compounds such as resveratrol have shown DPP4R inhibitory effect in an in vitro study. Resveratrol also showed potential to block the binding of ACE2 at the molecular level, which could reduce the virus entrance in the cell [48,136].

#### 3.3.3. Support on Decrease of Virus Replication

##### Increasing Intracellular Zinc Level and Synergistic Effect of Resveratrol (Box #12 and #16)

Zinc is a crucial element for normal development and function of cells mediating nonspecific immunity, such as neutrophils and NK cells; therefore, a good zinc status is recommended to support an effective immune function [3,68]. Preliminary studies showed that high concentration of intracellular zinc inhibited the replication of SARS coronavirus (SARS-CoV) and other RNA viruses, through inhibition of RNA polymerase [67]. Resveratrol can act synergistically with zinc, as it has been shown to increase the intracellular entrance of zinc in vitro [84].

##### TLR7 Activation/Boosting Interferon Type 1 Response (Box #17 and #19)

IFNs can inhibit SARS coronavirus replication and might then be valuable for COVID-19 treatment [137]. The activation of TLR7 by single-stranded viral RNA trapped within endosomes provides a key stimulus to type 1 IFN induction by RNA virus. Ferulic acid (and sodium ferulate) can induce heme oxygenase-1 (HO-1), which can account for the activation of TLR7 and stimulation of the type 1 IFN. Spirulina possesses a phycocyanobilin chromophore that mimics NAPDH-oxygenase, which could explain the antioxidant and anti-inflammatory effects of the cyanobacteria, as it inhibits the activity of unconjugated bilirubin. The ingestion of spirulina may also have a potential for boosting type 1 IFN response against RNA virus infection. N-acetylcysteine, selenium, and glucosamine might be expected to help to prevent and control RNA virus infections because they amplify the signaling functions of TLR7 and mitochondrial antiviral-signaling protein (MAVS) in type 1 IFN production [3].

#### 3.3.4. Support on Control of Hyperinflammation

##### Lymphocytes B Proliferation and Differentiation (Box #7)

Cholecalciferol possibly plays a role on the regulation (inhibition) of B cell proliferation and differentiation (B cells are recognized as key factors in inflammation, as they can secrete inflammatory cytokines) [68]. As cholecalciferol exhibits anti-inflammatory properties, and therefore could potentiate innate immunity while controlling the potentially harmful inflammatory response. This immunoregulatory effect could in turn prevent hyperinflammatory response caused by respiratory tract infections [74,76,77]. In fact, vitamin D3 may possibly decrease the IL-6 effect, which is a known marker of poor outcome in critically ill patients [138]. Evaluation of a large number of patients from several countries has demonstrated that cholecalciferol may reduce COVID-19 severity by a suppressive effect on the cytokines (storm) [78,139,140]. Indeed, a negative correlation between cholecalciferol serum levels and the number of cases of COVID-19, and also the number of deaths caused by COVID-19, has been observed [141,142,143]. Ascorbic acid can increase lymphocyte B and T proliferation and differentiation at a controlled rate [71].

##### Inhibition of TL4 (Box #11)

The innate immune system recognizes pathogen-associated molecular patterns (PAMPs) of viral intruders via pattern recognition receptors (PRRs), which includes the TLRs family. The TLR4-induced innate immunity can act favoring viral replication, and consequently lead to an excessive inflammatory response. In this sense, TLR4 inhibitors can play a role in alleviating the symptoms in viral infections. Resveratrol and ferulic acid can inhibit the TLR4 signaling pathway, which provides potential protection against tissue damage (including lung) coming from excessive inflammatory response [3]. There is also evidence suggesting that TF could modulate the response to the recognition of micro-organisms through the activation of TLR4-MD2 complex, which occurs through the MyD88-mediated NF-kB pathway [57].

##### Inflammatory Interleukine-6 Inhibition (Box #13 and #20)

Elevated IL-6 is a typical inflammatory finding in serum of patients with acute respiratory distresses, including COVID-19. The inhibition of IL-6 may then decrease the damage to lung tissue caused by the cytokine storm in patients with severe symptoms due to infections with coronaviruses [43,44].

TF have shown the potential to decrease IL-6 levels in vitro, which could in turn downregulate overstimulation of the immune system. This effect would bring potential benefits in a hyperinflammatory stage of coronavirus infections. They have also shown to stimulate the release of IL-10, a cytokine that inhibits Th2 cells, thus playing a role avoiding immune hyperresponsiveness and hyperinflammatory condition in COVID-19 [55,57]. In humans, spirulina supplementation in healthy elderly has shown to increase IL-2 (participates in T cells maturation) and decrease IL-6 [129].

##### Inhibition of TNF-α and NF-kB Activation (Box # 14, #15, # 18 and #21)

Nucleocapsid and spike proteins from coronaviruses promote NF-κB activation, which can possibly contribute to the hyperinflammation profile of COVID-19. Elderly patients generally display more inflammation, linked to a central role of NF-κB and a reduction in expression of IFN-β [126]. In fact, virus infections activate cellular transcription factors (e.g., IRF-3 and NF-κB), which in turn stimulate the expression of the IFN genes. The released IFN initiates a signaling cascade of the JAK/STAT pathway that results in activated transcription factors translocating to the nucleus [144]. Therefore, NF-κB inhibitors could be promising for prevention and adjuvant treatment of coronaviruses infections, especially in elderly people [126].

Miodesin^®^ can inhibit the release of cytokines (IL-1β, IL-6, IL-8, and TNF-α) and chemokines (CCL2, CCL3, and CCL5) and the expression of NF-κβ, inflammatory enzymes (COX-1, COX-2, PLA2, iNOS), and chemokines (CCL2, CCL3, and CCL5) [61]. This could be beneficial to COVID-19 patients to the extent that it can decrease the immune hyperresponsiveness and inflammation in respiratory condition, with the potential to control the cytokines storm in acute respiratory infections [59]. Cholecalciferol is also suggested to suppress cytokine storm in COVID-19 patients, and as a result reduce mortality [78]. Resveratrol can inhibit the production of inflammatory factors through the activation of Sirtuin 1 (Sirt1), which is an important deacetylase involved in numerous molecular events (metabolism, cancer, embryonic development and immunotolerance). One of the main substrates of Sirt1 is p65. The activation of Sirt1 by resveratrol can inhibit RelA acetylation, with consequent reduction of NF-kβ and decrease in the expression of TNF-α, IL-1, IL-6, metalloproteases (MMP-1 and MMP3), and COX-2 [79,81,82].

Among the potential pharmacological effects of ferulic acid figures the decrease of the serological concentration of TNF-α and IL-1β, the suppression in TLR4 expression and the reduced activation of MAPK and NF-κB [145,146].

Some studies suggest that TF could regulate (inhibit) TNF-α production through the inhibition of the NF-κB [57,147].

##### Interleukin-7 Regulation and Lymphopoietic Stimulation

Interleukin-7 (IL-7) plays an important role in the immune system homeostasis and maintenance of health span [148]. IL-7 is a cytokine that has several effects on the hematopoietic and immunologic systems, and is best known for its role in supporting B- and T-lymphopoiesis [149]. IL-7 has been shown to stimulate in vitro T-cell development and reactivity, and then could possibly be used to boost development and function of such cells in diverse clinical settings, including infectious diseases [150]. All of the main CD4 T cell subgroups, including CD4^+^ immature, memory, and Th17 cells, depend on this cytokine for peripheral homeostasis. Interestingly, IL-7 can be found increased in the serum of patients having either mild/moderate or severe forms of COVID-19, which possibly represents the attempts of the immune system to reverse lymphopenia and T cell exhaustion [151,152].

Currently, scientists in the United Kingdom are testing (in a multicenter, Phase 2 clinical trial) the ability of IL-7 to produce an immune reconstitution of COVID-19′s severe patients and observing possible association with their clinical improvement [153]. Additionally, it is hypothesized that the bovine dialyzable leukocyte extract (TF) could help to increase IL-7 levels, based on the findings from a study with breast cancer patients. In this study, patients in chemotherapy and using TF were able to maintain the lymphocytes population within the reference range, while the group with only chemotherapy had this population dropped below such range [154].

#### 3.3.5. Support on Reduction of Oxidative Stress

SARS-CoV-2 can trigger a recurrent mechanism for viral infections: it activates a nonspecific, pro-oxidant response from macrophages through TLR stimulation that results in TNF-α activation of NADPH which, in turn, mediates ROS production. Macrophages also produce ferritin to protect themselves from ROS. ROS oxidizes hemoglobin to methemoglobin, and it induces latent chronic hemolysis. Therefore, the erythrocytes leak lactate dehydrogenase. In addition to that, ROS can damage the endothelial cell membranes, which causes the production of NO* radicals that activate Ca^2+^ channels through S-nitrosylation in myocytes leading to vasoconstriction. In a healthy condition, the molecules of the antioxidant system are in reduced form to neutralize the ROS. Throughout pathological conditions, the antioxidant molecules become oxidized. The transformation of molecules from their oxidized to reduced form requires regular uptake of exogenous antioxidants since humans cannot accumulate them [155]. In this light, antioxidants supplements could potentially benefit patients with COVID-19.

The relationship between the use of ACE2 by the SARS-CoV-2 to enter the cell and oxidative, inflammatory, and thrombotic events has also been investigated recently. In severe COVID-19 patients, the interference in systemic (and not only lung) ACE2 can lead to an increase on angiotensin II levels. This will account for ROS release and dysregulation of endothelial nitric oxide synthase (eNOS) and NADPH oxidase 2 (NOX2) (antioxidant and vasodilatory signals), which will then activate the complement system. This mechanism can partially explain the link between the oxidative stress and the inflammation and thrombotic events seen in COVID-19 [156].

Glutathione in its reduced form (GSH) and glutathione peroxidase (GPx) are the most essential antioxidants, both intra- and extracellular. They neutralize ROS and convert them to nontoxic products (H_2_O) [155]. Phase 2 inductive nutraceuticals as ferulic acid and resveratrol induce various peroxidase enzymes (enzymes that neutralize hydrogen peroxidase, a ROS) and promote synthesis of glutathione. Glutathione production can also be promoted by administration of N-acetylcysteine. The utility of N-acetylcysteine in the elderly might reflect the fact that plasma cysteine levels and cellular glutathione levels tend to decline with advancing age. Since selenium is an essential cofactor for certain peroxidases, selenium supplementation might also be appropriate in this context [3]. Besides, other nutraceuticals with antioxidant properties such as ascorbic acid, spirulina and a blend of herbal extracts including *Uncaria tomentosa*, *Endopleura uchi* and *Haematoccocus pluvialis* could also contribute to reduce the oxidative stress [63,64,71,129,157].

#### 3.3.6. Potential Antithrombotic Effect

SARS-CoV2 may induce intravascular pulmonary thrombosis, which can result in the rapid worsening of the patient’s clinical condition. As previously stated, the inflammatory and thrombotic events observed in COVID-19 appear to be linked to an uncontrolled and systemic oxidative stress linked to the increased angiotensin II levels [156]. In this perspective, antithrombotic/coagulation adjuvant supplements could be beneficial by their impact on the inflammation observed, hence protecting the vessel wall [158]. In addition, the complement system seems to be associated with microvascular injury and thrombosis in severe COVID-19 patients. A preliminary study showed that some COVID-19 patients have presented purpuric skin lesions and pauci-inflammatory thrombogenic vasculopathy, with deposition of C5b-9 and C4d in skin. In addition, there appears to be colocalization of COVID-19 spike glycoproteins with C4d and C5b-9 in the interalveolar septa and the cutaneous microvasculature. Thus, COVID-19 may possibly correspond to a type of microvascular injury syndrome mediated by activation of complement pathways and an associated procoagulant state [156].

There is some evidence related to a potential antithrombotic effect of resveratrol due to the attenuation of the activation of thrombosis-related markers by H_2_O_2_ via SIRT_1_ signaling [159]. Indeed, polyphenols compounds have been shown to be able to modulate the function of the different cellular components involved in the process of thrombosis in different systems. Interference with arachidonic acid metabolism in both platelets and leukocytes have been reported in vitro, which can be a signal that it inhibits platelet aggregation and reduces synthesis of prothrombotic and proinflammatory mediators. Polyphenols are also known for their capacity to downregulate the expression of adhesive molecules and tissue factor activity induced by cytokines. This possibly results in functional modulation of cell–cell interactions and procoagulant activities [160]. Ferulic acid also has an antithrombotic effect and some studies have suggested its role in thrombotic diseases, such as cardiovascular dysfunction, pulmonary thromboembolism and deep vein thrombosis. It can regulate the blood coagulation function in two aspects: inhibiting platelet aggregation and protecting endotheliocyte [161]. Finally, one tetracyclic alkaloid of *Uncaria tomentosa* could have an effect on thrombosis, as it has been reported as a potent inhibitor of platelet aggregation and venous thrombosis [111].

#### 3.3.7. Potential Protection of Endothelial Barrier

SARS-CoV-2 can infect endothelial cells and cause systemic vascular endotheliitis, which, in turn, can lead to vasoconstriction, with subsequent organ ischemia (kidney, lung, heart, liver, and brain), oxidative stress, inflammation with associated edema, and a prothrombotic state. Endothelial dysfunction is also an important factor for atherosclerosis [162].

To counteract the SARS-CoV-2-induced endotheliitis, endothelial barrier protectors such as resveratrol, vitamin D_3_, silicon, vitamin C, and astaxanthin could play a role in ameliorating the endothelial health [125,163,164,165,166,167,168].

Resveratrol attenuates endothelial inflammation by protecting cells from stressful conditions, which is mediated through the activation of the signaling cAMP-PRKA-AMPK-SIRT1 pathway. Despite its low systemic bioavailability, it accumulates in the endothelial cells [165]. Its endothelial effects also seem to be mediated by activation of nuclear-E2-related factor-2 (NRF2) [163].

Vitamin D_3_ protects the endothelium from oxygen peroxide (H_2_O_2_) injury, and through modulation between apoptosis and autophagy. It has been seen that vascular of proinflammatory transcription factor NF-κB and endothelial proinflammatory cytokine IL-6 was also higher in vitamin-D_3_-deficient patients. Vitamin D_3_ has also been shown to modulate (in vitro) the levels of systemic inflammatory cytokines, such as TNF-α and IL-6, as well as inhibit lipopolysaccharide (LPS) induced activation. One clinical study has also shown that vitamin D_3_ can be inversely associated with levels of 3-nitrotyrosine and soluble vascular cell adhesion molecule-1 (sVCAM-1), one indicative of decreased nitrosative stress and endothelial activation [164].

Vitamin C participates in important functions in the support of endothelial cells. These functions include increasing synthesis and deposition of type IV collagen in the basement membrane, stimulating endothelial proliferation, inhibiting apoptosis, scavenging radical species, and sparing cell-derived nitric oxide to help modulate blood flow [168].

One of the constituents from Miodesin^®^ (*Uncaria tomentosa*) is rich in oxindole alkaloids and polyphenols, including phenolic acids and proanthocyanidins (procyanidins, flavalignans, and propelargonidins), which show positive correlation with the antioxidant capacity of the plant species. *Uncaria tomentosa* pentacyclic oxindole alkaloids stimulate endothelial cells (observed in vitro) to produce a lymphocyte-proliferation-regulating factor. Proanthocyanidins have also shown antithrombotic properties associated with endothelial protection and inhibition of inflammatory cells adhesion, as it causes a decrease in the expression of P-selectin (thus inhibiting leukocytes and thrombosis) [63,64,165]. Another constituent from Miodesin^®^ (astaxanthin from *Haematococcus pluvialis* extract) is also an endothelial protector due its antioxidant properties: it has been demonstrated to inhibit intracellular induced stress in human endothelial cells without any cytotoxicity and modification of the cell morphology [125].

Finally, the antiatherogenic effect of oral silicon has been reported [138,166,167,169,170,171]. Although the mechanism of Si has not been well clarified, one study has found that Si modifies the characteristics of endothelial relaxants and attenuates smooth muscle cell responsiveness to nitric oxide (NO). The findings demonstrated that the orally administration of Si for a short period (eight days) was able to change both characteristics of endothelial dilators and smooth muscle cell response to them. Si seemed to reduce NO generation without distributing endothelial functions, because acetylcholine-elicited relaxation in Si-exposed group was higher than those of controls [167].

### 3.4. Safety Considerations

**Imuno TF^®^**

- *Safety:* Transfer factors have been used in different applications since the 1950s, with no reported adverse effects. Additionally, long-term oral administration has been reported as safe [55,172]. Transfer factors can be considered as possibly safe when used for up to two years in adults [173].
- *Contraindications:* In case of use of immunosuppressants, they have antagonistic effects (Imuno TF® regulates the immune system, increasing Th1 response)”. There is not enough information about the use of the transfer factor during pregnancy and breastfeeding. Avoid use during this period [173].
- *Drug interaction:* The effects of transfer factors can be reduced with the use of corticosteroids.
- *Adverse effects:* Rare. Occasionally, when the patient starts TF treatment, typical flu symptoms, fever episode, nausea, and gastrointestinal symptoms may occur. These symptoms are usually classified as Jarisch–Herxheimer reactions and are probably related to a direct reaction of TF in the intestine or systemic pathogens [174].

**Miodesin^®^**

- *Safety:* Miodesin^®^ does not induce changes in DNA [61]. *Uncaria tomentosa* (Willd.) DC. and *Haematococcus pluvialis* (astaxanthin esters) are classified by the Dietary Supplements Information Expert Committee (DSI-EC) of the United States Pharmacopeial Convention as Class A, which indicates that the available evidence does not indicate a serious risk to health – this substance has a monograph in United States Pharmacopeia and National Formulary (USP–NF). Preliminary studies with *Endopleura uchi* (Huber) Cuatrec. (*Humiriaceae*) did not reveal any toxicity [175,176].
- *Contraindications:* Active ingredients included in Miodesin^®^ are contraindicated for patients with rheumatism and patients who will undergo or have had an organ transplantation. The use of Miodesin^®^ during pregnancy and lactation should be discussed with the prescriber [111].
- *Drug interaction:* Miodesin^®^ is contraindicated for concomitant use with immunosuppressants due to its immunostimulant effect. Drug interactions may occur with warfarin, estrogens, theophylline, ginger and drugs metabolized by the cytochrome P-450 route. In patients taking these medicines, Miodesin^®^ should be administered under medical supervision [177]. Miodesin^®^ may also potentiate the action of antihypertensive drugs [178].
- *Adverse effects:* active ingredients of Miodesin^®^ may cause fatigue, fever, diarrhea and constipation [177].

**Zinc orotate or gluconate**

- *Safety:* United States Pharmacopeia provides monograph for this substance as a pharmaceutical ingredient (as gluconate).
- *Contraindications:* Iron and copper deficiency [179].
- *Drug interaction:* concurrent administration of zinc salts may diminish the absorption of tetracycline [180]. Large doses inhibit iron and copper absorption [179]. Amiloride reduces zinc excretion, leading to its accumulation in the body [181]. Consumption of fiber-containing foods inhibit absorption of zinc, then take the medicine an hour before, or two hours after, consumption of food high in fiber [182].
- *Adverse effects:* Side effects of zinc salts are abdominal pain, dyspepsia and diarrhea [183]. No effects have been reported for fertility, pregnancy and lactation [184]. Zinc accumulation in the body could lead to toxic side effects, such as metallic taste sensation, vomiting, and stomach problems [185].

**Selenium yeast**

- *Safety:* The reference dose for selenium (70 kg adult) is defined by EPA as 350 µg, [186] and the European Food Safety Authority (EFSA) provided safety evaluation on selenium yeast (safe up to 0.2 mg Se/kg) [187].
- *Contraindications:* In cases of selenium poisoning or hypersensitivity to products containing selenium. Pregnancy: there are no data from the use of selenium in pregnant women; selenium is excreted in human milk, but at therapeutic doses, no effects are anticipated in newborn/lactating infants. Selenium can be used during lactation. There are no data on fertility with the use of selenium in humans; selenium did not affect male fertility in rats and the effects of selenium on female fertility in rodents were only observed at very high doses. In general, doses to correct selenium deficiency are not expected to have adverse effects on fertility [188].
- *Drug interaction:* Major interaction with the drug eltrombopag; do not use both substances simultaneously [189]. Selenium is generally incompatible with high concentrations of ascorbic acid (reduction of selenite to elemental selenium which is not soluble and not available as a nutritional source of selenium) [188].
- *Adverse effects:* Gastrointestinal upset. Very high selenium dosages (above 850 µg daily) are known to cause selenium toxicity, whose signs include depression, nervousness, emotional instability, nausea, vomiting, and in some cases loss of hair and fingernails [185].

**Vitamin D_3_**

- *Safety:* United States Pharmacopeia has provided a monograph for this substance as a pharmaceutical ingredient. The reports of vitamin D toxicity show that hypercalcemia involve serum 25(OH)D concentrations when it is greatly above 200 nmol/L. To achieve this level, a daily intake higher than 40,000 IU would be required—then, this value could be considered as the lowest observed adverse effect level (LOAEL) for vitamin D [190].
- *Contraindications:* Treatment of pregnant women with high-dose vitamin D (>4000 IU/day) is contraindicated [191]. People with sarcoidosis or hyperparathyroidism should never take vitamin D without first consulting a physician [192].
- *Drug interaction:* Vitamin D is a chemical structure similar to calcitriol; do not use medications containing calcitriol while using vitamin D. Vitamin D_3_ may interfere with cholesterol laboratory tests, possibly causing false test results [191].
- *Adverse effects:* Vitamin D at normal doses usually has no side effects. At high doses, it can occur gastrointestinal (nausea and vomiting), metabolic (hypercalcemia), renal (hypercalciuria) and dermatological (pruritus, urticaria) effects [191].

**Vitamin C**

- *Safety:* United States Pharmacopeia has provided a monograph for this substance as a pharmaceutical ingredient.
- *Contraindications:* G6PDH deficiency [193]. Nephrolithiasis patients or with history of nephrolithiasis; hyperoxaluria; patients with severe kidney failure or kidney failure; hemochromatosis [194]. There are no controlled studies regarding the use of ascorbic acid in pregnant women; ingestion of high doses of the vitamin in pregnant women can produce scurvy in the newborn. Ascorbic acid is excreted in breast milk; there is insufficient data on the effects of ascorbic acid supplementation in newborns. The product should only be administered during pregnancy or lactation when considered essential by the doctor. The recommended dose should not be exceeded, as chronic overdose can be harmful to the fetus and newborn. There is no evidence to suggest that normal endogenous levels of ascorbic acid cause adverse reproductive effects in humans [194].
- *Drug interaction:* Oral anticoagulants such as warfarin and acenocoumarol: their action could be modified by ascorbic acid in large doses. Deferoxamine: concurrent use with high doses of ascorbic acid may potentiate iron tissue toxicity, with impaired cardiac function, causing cardiac decompensation; ascorbic acid should not be administered during the first month of deferoxamine treatment. Cyanocobalamin (vitamin B_12_): ascorbic acid in large doses may reduce the amounts of cyanocobalamin available in serum and reserves; ascorbic acid is recommended to be administered at least 2 h after meals. Indinavir (protease inhibitors): high doses of ascorbic acid significantly decrease the plasma concentration of indinavir, with a probable reduction in its efficacy. Cyclosporine: limited data suggests that antioxidant supplements like ascorbic acid may lower cyclosporine blood levels. Disulfiram: chronic or high doses of ascorbic acid can interfere with the effectiveness of disulfiram. Iron: ascorbic acid can increase iron absorption, especially in people with iron deficiency; small incremental increases in iron may be important in subjects with conditions such as hereditary hemochromatosis or in subjects who are heterozygous for this condition, as it may exacerbate iron overload [194].
- *Adverse effects:* Metabolism and nutrition disorders: in especially predisposed patients, gouty arthritis may occur, and uric acid stones may form. Nervous system disorders: headache, insomnia. Gastrointestinal disorders: diarrhea, nausea, vomiting, abdominal and gastrointestinal pain. Renal and urinary disorders: the administration of ascorbic acid in individuals predisposed to increased stone formation has been associated with the production of oxalate, urate or cystine stones, or precipitation of drugs in the urinary tract; subjects with the highest risks are those with renal impairment [194].

**Ferulic acid**

- *Safety:* As an organic compound derived from food, ferulic acid is presumed to be safe, despite the lack of toxicological studies [195]. Its oral LD50 in rats is 2445 mg/kg body weight [196].
- *Contraindications:* The safety of ferulic acid in children, pregnant women, or nursing mothers has not been established, therefore precaution is to be taken for these groups [195].
- *Drug interaction:* One animal study (mice) showed that ferulic acid increases the blood levels of the anticoagulant clopidogrel, increasing the risk of bleeding and bruising, but this was yet not confirmed in humans [197].
- Adverse effects: Not currently reported for humans by oral route.

**Resveratrol**

- *Safety:* EFSA has provided safety evaluation on resveratrol (safe up to 150 mg/day) [198].
- *Contraindications:* Resveratrol might slow blood clotting and increase the risk of bleeding in people with bleeding disorders. Resveratrol might have estrogen-like actions—if the patient has any condition that might be made worse by exposure to estrogen, use is not recommended [199].
- *Drug interaction:* Resveratrol may interact with carbamazepine and other substrates of CYP3A4 [200].
- *Adverse effects:* Not currently reported [199].

**Spirulina**

- *Safety: Spirulina maxima* is classified by the Dietary Supplements Information Expert Committee (DSI-EC) of the United States Pharmacopeial Convention as Class A, which indicates that the available evidence does not indicate a serious risk to health, and permits this substance has a monograph in United States Pharmacopeia and National Formulary (USP–NF) [201].
- *Contraindications:* Phenylketonuria (cyanobacteria may contain the amino acid phenylalanine) [202]. Information regarding safety and efficacy in pregnancy and lactation are currently not available, then spirulina should be avoided during this period [203,204]. Patients with autoimmune disorders may present adverse reactions when consuming immunostimulatory herbal preparations [205].
- *Drug interaction:* No interaction currently documented *in vivo*. Antiplatelet action was demonstrated in vitro [206].
- *Adverse effects:* No major effects have been reported, only isolated cases of immunoblistering [207], rhabdomyolysis [208] and hepatotoxicity (possibly due to the presence of microcystins and anatoxin-a) [209,210,211].

**N-acetylcysteine**

- *Safety:* United States Pharmacopeia provides monograph for this substance as a pharmaceutical ingredient.
- *Contraindications:* This drug crosses the placenta and was measurable in the serum of infants. Use is not recommended during pregnancy unless clearly needed [212].
- *Drug interaction*: May alter the absorption of inhaled human insulin [212].
- *Adverse effects:* The most common adverse events are anaphylactoid reaction, nausea, vomiting, flushing, and skin rash [212].

**Glucosamine sulfate potassium hydrochloride**

- *Safety:* The oral LD50 for glucosamine has been estimated to be >8000 mg/kg body weight in rats and mice and >6000 mg/kg in rabbits [213].
- *Contraindications:* There is not enough data showing if glucosamine sulfate is safe to be used during pregnancy or while breast-feeding. Avoid use during this period. There are preliminary reports suggesting that glucosamine sulfate can increase insulin levels, which could cause an increase in cholesterol—then, the cholesterol levels should be monitored if the patient is taking glucosamine and has high cholesterol [214].
- Drug interaction: Warfarin (increases the effect, slowing blood clotting) and antineoplastic drugs such as etoposide, teniposide and doxorubicin (antagonist effect on cell division) [214].
- *Adverse effects:* Glucosamine sulfate can cause some mild side effects including nausea, heartburn, diarrhea, and constipation. Rare side effects are drowsiness, skin reactions, and headache [214].

**Maltodextrin-Stabilized Orthosilicic Acid**

- *Safety:* EFSA has provided a safety evaluation on orthosilicic acid from different sources (from 5 mg Si/day to 18 mg Si/day, depending on the source) [215,216].
- *Contraindications:* In case of silicon poisoning or hypersensitivity to products containing silicon [217]. There is no information available on the use of silicon during pregnancy or while breastfeeding. Avoid use during this period.
- *Drug interaction:* Silicon has no known severe, serious, moderate, or mild interactions with other drugs [217].
- *Adverse effects:* There are no known side effects associated with using silicon up to date; silicon is present in neurofibrillary tangles in Alzheimer’s disease [217].

## 4. Conclusions

In order to control the SARS-CoV-2 infection in the human body, we underline seven main mechanisms that could be addressed to impact the COVID-19 outcome: (i) regulation of the immune system; (ii) avoidance of virus entrance in the cell; (iii) decrease of virus replication; (iv) control of hyperinflammation; (v) reduction of oxidative stress; (vi) antithrombotic effect; and (vii) protection of the endothelial barrier. In this review, we have evaluated the possible roles that twelve ingredients may have on such mechanism: Imuno TF^®^ (transfer factors), Miodesin^®^ (*Uncaria tomentosa*, *Endopleura uchi* and *Haematoccocus pluvialis*), zinc, selenium, ascorbic acid, cholecalciferol, trans-resveratrol, ferulic acid, spirulina, N-acetylcysteine, glucosamine sulfate potassium hydrochloride, and maltodextrin-stabilized orthosilicic acid (SiliciuMax^®^). All these agents can act on some of the referred mechanism of the COVID-19. Thus, they could provide a beneficial role in the prevention or improvement of the COVID-19-associated symptoms. The present review article creates the hypothesis that the combination of agents proposed here can play a role in the recovery of COVID-19 patients, although an evaluation in controlled, randomized clinical trials is now necessary to confirm the therapeutic potential of the therapeutic agents described.

## Figures and Tables

**Table 1:** Ingredients that could play a role in the prevention and/or support treatment of the symptomatology associated with COVID-19 and suggested quantity (daily intake). The suggested posology is to divide this daily intake into three doses, to be taken every 8 h.

Ingredients	Suggested Quantity	Action and Use (Key Points)
Transfer factors (oligo- and polypeptides from porcine spleen, ultrafiltered at < 10 kDa; Imuno TF^®^)	100 mg	Immunoregulator. It can enhance the antigenic stimulus, making CD4^+^ Th1 cells to produce IFN-γ, IL-1 and TNF-α. It can also modulate the response to the recognition of microorganism via activation of TLR4-MD2 complex and can increase innate defense against invasive microbial/viral infection and inflammation in not previously immunized patients. It has shown a potential to decrease the level of proinflammatory IL-6, and to stimulate the release of IL-10, a cytokine that inhibits Th2 cells, thus playing a role avoiding immune hyperresponsiveness and hyperinflammatory condition [55,56,57,58].
Anti-inflammatory blend of natural extracts from *Uncaria tomentosa*, *Endopleura uchi* and *Haematoccocus pluvialis* (Miodesin^®^)	800 mg	Presents anti-inflammatory properties, as it inhibits the release of cytokines (IL-1β, IL-6, IL-8, and TNF-α) and chemokines (CCL2, CCL3, and CCL5) and the expression of NF-κβ, inflammatory enzymes (COX-1, COX-2, PLA2, iNOS), and chemokines (CCL2, CCL3, and CCL5) [59,60,61]. The alkaloidal fraction of its composition (oxindoles) can play a role in immunoregulation, while triterpenoid alkaloids and quinovic acid glycosides present in the product may inhibit some DNA and RNA viruses [62]. It can also contribute to reducing the oxidative stress and to enhance phagocytosis (glycosides) [63,64].
Zinc (as orotate or gluconate)	60 mg	Supports the effective function and proliferation of numerous immune cells, such as neutrophils and NK cells, as well as the humoral immune response [65,66]. Increasing concentration of intracellular zinc inhibits the replication of SARS coronavirus (SARS-CoV) and other viruses [67].
Selenium yeast	48 mg (equivalent to 96 µg of Se)	Important element for optimal innate and adaptative immune response, as it stimulates T helper lymphocytes, cytotoxic T and NK cells, and macrophage phagocytosis. Deficiency of selenium induces impairment of the host’s immune system and mutation of benign variants of RNA viruses to virulence. It also has potential to help in the prevention and control of RNA viruses by amplifying the signaling functions of TLR7 [3,68,69].
Ascorbic acid (Vitamin C)	300 mg	Remarkable antioxidant [70]. It can improve the functionality of immune system, reducing the severity of infections and its symptoms through the enhancement of T-cell and NK cell function and proliferation [68,71,72,73].
Cholecalciferol (Vitamin D3)	20,000 IU	Possesses immunoregulatory effect that can prevent hyperinflammatory response caused by respiratory tract infections, as it increases lymphocytes T and B proliferation and maturation, as well as immunoglobulins production [68,74,75,76]. It presents antiviral and antibacterial effects [77]. The restoration of Vitamin D normal levels reduces the rates of C-reactive protein, which is estimated to reduce in nearly 15% the severe COVID-19 cases [78].
*Trans*-resveratrol	90 mg	Potent antioxidant. It can activate NK cells, suppress TLR4 and proinflammatory genes’ expression, and reduce NF-kB, with decrease in the expression of TNF-α, IL-1, IL-6, metalloproteases (MMP-1 and MMP3) and Cox-2 [79,80,81,82]. It also presents potential to reduce virus entrance in the cells, and can act synergistically with zinc to decrease the virus replication rate [83,84].
Ferulic acid	480 mg	Participates in the activation of TLR7, induction of heme oxygenase-1 (HO-1) activity, and prevention and control of RNA virus infections by amplifying the signaling functions of TLR7 and MAVS [3].
Spirulina (*Spirulina máxima*)	800 mg	Possesses a direct effect on both innate (activation of macrophage and NK cells) and specific immunities (regulation of T cells and increased production of antibodies) [68,85,86]. Phycocyanobilins present in spirulina may have potential for boosting type 1 IFN response in the context of RNA virus infection [3].
N-Acetylcysteine	560 mg	Helps to prevent and control RNA virus infections by amplifying functions of TLR7 and mitochondrial antiviral-signaling protein (MAVS) in evoking type 1 IFN production [3].
Glucosamine sulfate potassium chloride	610 mg	May upregulate MAVS activation, therefore it may play a role in the prevention and the control of RNA virus infections [3].
Maltodextrin-stabilized orthosilicic acid (SiliciuMax^®^)	400 mg (equivalent to ≡ 6 mg of Si)	Silicon can potentially provide a net increase in circulating lymphocytes and immunoglobulins (especially IgG) [87].

## Glossary

**C3a and C5a**	complement components C3a and C5a; anaphylatoxins
**CD4^+^**	T helper/amplifier cells
**CD8^+^**	cytotoxic subpopulation of T cells
**CLR**	C-type lectin receptors
**CoV**	a family of viruses in which the SARS-CoV-2 strain is included
**COVID-19**	coronavirus disease 2019
**DPP4**	dipeptidyl peptidase-4
**dsRNA**	double stranded RNA viruses
**IFN**	interferon (IFN-α, IFN-γ)
**Ig**	immunoglobulin (IgA, IgD, IgE, IgG, IgM)
**IKK**	IκB kinase
**IL**	interleukin (IL-1, IL-2, IL-6, IL-8, IL-21, etc)
**IRAK4**	interleukin-1 receptor-associated kinase-4
**IRFs**	interferon regulatory factors
**JAK-STAT**	Janus kinase/signal transducers and activators of transcription
**MAD-5**	melanoma differentiation-associated protein 5
**MAPK**	mitogen-activated protein kinase
**MAVS**	mitochondrial antiviral-signaling protein
**MCP-1**	membrane cofactor protein 1
**MyD88**	myeloid differentiation primary response 88
**NEMO**	nuclear factor-kappa B essential modulator
**NF-** **κ** **B**	nuclear factor-κB
**NK cells**	natural killer cells
**PAMPs**	pathogen-associated molecular patterns
**PKCs**	protein kinase C
**PPRs**	pattern recognition receptors
**RIG-I**	retinoic acid-inducible gene I
**S protein**	spike protein
**SARS-CoV-2**	severe acute respiratory syndrome coronavirus 2
**ssRNA**	single stranded RNA viruses
**Th cells**	T helper cells (Th1, Th2, Th17)
**TIRAP**	toll-interleukin 1 receptor (TIR) domain-containing adapter protein
**TLR**	toll-like receptors (TLR3, TLR4, TLR7)
**TNF**	tumor necrosis factor (TNF-α, TNF-β)
**TRAF6**	tumor necrosis factor receptor associated factor 6
**TRAM**	TRIF-related adaptor molecule
**TRIF**	TIR-domain-containing adapter-inducing interferon-β
